# The Two-Tier Problem

**DOI:** 10.1163/17455243-21050021

**Published:** 2024-10-15

**Authors:** Dominic JC Wilkinson

**Affiliations:** Uehiro Oxford Institute, Faculty of Philosophy, University of Oxford, Oxford, United Kingdom; John Radcliffe Hospital, Oxford, United Kingdom; Murdoch Children’s Research Institute, Melbourne, Australia; Centre for Biomedical Ethics, National University of Singapore; Yong Loo Lin School of Medicine, Singapore, Singapore

**Keywords:** ethics, non-identity, reproduction, harm, genetics

## Abstract

A number of philosophers argue for a Two-Tier View: that there is some difference between individual-affecting and non-individual-affecting choices. But it is challenging to know the degree of moral difference, and to determine for some cases into which category they fall. I refer to this as the “Two Tier Problem.”

In this paper, I develop and defend a “Two-Tier Deontic View.” On that view, the higher tier applies to a subset of individual-affecting cases. We have stronger reason to bring about an individual-affecting rather than a non-individual-affecting benefit, but only in cases where we have agent-relative duties to the individuals so affected. In other cases (and I argue that this applies to most policy decisions affecting reproduction) there is no moral difference between individual- and non-individual-affecting choices.

## 1 Introduction

A decision maker is considering two different ways of preventing a serious genetic problem.

**Preventing a Genetic Problem**

Option 1 (**Gene editing**): This involves a newly developed form of germline gene editing. Couples who are carriers of a genetic disorder, who have undertaken pre-implantation genetic diagnosis and have no unaffected viable embryos would be offered the option of gene therapy rather than implanting an unmodified but affected embryo.

Option 2 (**Embryo Selection**): In this option, couples who are carriers of a genetic disorder would be offered the option of in-vitro fertilization with pre-implantation genetic diagnosis. Embryos without the genetic disorder would be implanted rather than embryos with the disorder.

Imagine that our decision maker knows that the second option would be twice as likely to result in a child without the genetic disorder. Which option should they choose?

Another decision maker is considering ways of reducing the number of children affected by serious birth defects arising as a complication of a commonly used medicine.^1^ They are aware of two different ways of doing this.

**Preventing Birth Defects**

Option 1 (**Pregnancy detection**): A program is being considered which would identify women due to start on the medicine who are pregnant and use a different medication instead. It would also identify early pregnancy in women taking the medicine and arrange for them to switch over to an alternative medicine at an early stage of gestation. This program would mean that some children are prevented from having birth defects.

Option 2 (**Pregnancy prevention**): A program is being considered which would require women taking the medicine to use highly effective forms of contraception. This program would mean that fewer children are born with birth defects.^2^

In this case, imagine that our decision maker is aware that the pregnancy prevention program would prevent twice as many cases of the birth defects. Which option should they choose?

Although we might be tempted to simply choose the most effective option, as famously observed by Derek Parfit, these options differ in a potentially morally important way. One of our natural ways of explaining what is wrong with a choice is that it would be *worse* for the people affected by it. But if our decision leads to a different person being born (as occurs in some of the options above) we cannot say this. Parfit called this the “non-identity problem”^3^ and described individual cases as well as population-level versions, like **Preventing Birth Defects**. If we make certain choices, future people’s lives will be shorter, more unhappy, and less healthy. But those individuals may not be worse off, since had we made different choices, *other* individuals would have existed instead.

As I will discuss in more detail shortly, one attractive response to the non-identity problem is to suggest that both types of choices matter morally, but that choices that affect someone for the better or worse matter more.

If the options were equally effective, this might be significant in terms of either benefits or potential harms. For example, a fertility specialist may be tempted to offer gene editing rather than embryo selection (or a prospective parent might prefer that option) because this will *benefit* specific future children. Douglas and Devolder call this “the Benefit Argument” in favor of gene editing.^4^ Along similar lines, a public health policymaker might choose to fund *pregnancy detection* over *pregnancy prevention* because if she does not do so, future children affected by malformations will have been harmed.^5^

Yet, as imagined in the cases above, in many situations, these options will not be equally effective. They may prevent different numbers of cases of future illness, or they may be more costly, or in some cases they may be associated with additional complications. In that case, we need to weigh up the relative importance of these different types of choice.

That leads to a practical challenge, which I will call:

**The Two-Tier Problem:** The problem of deciding between individual-affecting and non-individual-affecting options, where those options will both affect the levels of future wellbeing but to different degrees.

In this paper, I aim to explore how, if we are drawn to a Two Tier View, we might respond to this problem. I first set out the relevant key concepts and alternative views. I then detail a series of epistemic and normative problems for applying the Two Tier View. In part in response to these problems, I develop and defend a “Two-Tier Deontic View.” On such a view, we have stronger reason to cause an individual-affecting rather than a non-individual-affecting benefit, but only in cases where (and to the degree that) agents have agent-relative ethical duties to the individuals so affected. I consider a range of potential objections. I argue that in policy decisions such as **Preventing Birth Defects**, the difficulties in applying a Two-Tier View are not necessarily a problem, since decision makers do not have duties to the individuals who will be conceived. This means that there is, in effect, no moral difference and we are justified in choosing the most effective alternative. However, in other cases, decision makers (for example parents) have a stronger moral reason to make an individual-affecting choice, even where this will lead to a worse outcome overall.

### 1.1 Terminology

The types of case in which the non-identity problem arises are sometimes referred to as:

***Non-Individual Affecting (NInA, non-identity)***: Cases in which different future individuals will exist depending on what we decide. For example:

Choice 1: A exists with wellbeing W_1_.

Choice 2: B exists with wellbeing W_2_.

The second options in **Preventing a Genetic Problem** and **Preventing Birth Defects** are both non-individual affecting. A related term – non-individual-affecting reasons – are the reasons that we have to choose in NInA cases.

To clarify, these cases will obviously “affect” some future individuals in the sense that choices will lead to those individuals existing. If someone can be benefited by coming into existence, we might say that these cases confer a non-comparative benefit on the future individual, A or B, who exists.^6^ But we cannot say in NInA cases that these individuals are better or worse off than they would have been had we made a different choice.

These can be contrasted with:

***Individual Affecting (InA)*:** Cases in which the same individuals exist or will exist, regardless of the decision that we make. For example:

Choice 1: A exists with wellbeing W_1_.

Choice 2: A exists with wellbeing W_2_.

The first options in the cases at the start of this paper, are both individual affecting. Again, we can refer to the reasons why we have to act in such cases as InA reasons.

One point to note: Parfit originally referred to “person affecting” reasons in relation to these cases. But it is better to avoid reference to ‘persons,’ since depending on the time point of intervention (and the capacities and nature of the individuals), they may or may not be ‘persons.’^7^

### 1.2 Assumptions

Before proceeding, I should set out some key assumptions. A standard way of discussing harms is through counterfactual comparisons. Someone is harmed if they are worse off than they would otherwise have been. I will assume such a counterfactual account of harms for this paper. Some authors propose or defend non-counterfactual accounts of harm.^8^ I do not propose to enter into debate about whether these are plausible. But for those who defend non-counterfactual harms, similar questions (and a similar approach to them) might arise. How should we compare counterfactual with non-counterfactual harms? Are there more reasons to avoid the former than the latter, (and if so, how much)?

Next: some health conditions or states might be so bad that the individual’s life is not worth living. In such cases, it would be arguably wrong to make choices that lead to them existing, even if the alternative is non-existence. The cases I discuss in this paper should be assumed to be associated with wellbeing decrements that are not this severe, i.e., are compatible with a life-worth living.

I should note two further complications that I will largely set aside. Many NInA choices will *also* affect other individuals. For example, they may affect parents, or they may affect wider society. For the sake of the cases in this paper, we should assume that these effects on other people are equivalent between the InA and NInA choices that we are making. Finally, some NInA choices will affect the number of future people who will come into existence. Parfit referred to the latter as “different number choices.”^9^ There are important but difficult questions about how to think about the morality of these decisions. I will focus on “same number choices” for this paper.

## 2 The Some-difference or Two-Tier View

In thinking about the types of case described above, Parfit contrasted two different approaches.

One view is that morality is confined to what makes individuals better or worse off.^10^ We could call this:

**The Great Difference View**:^11^ There is *no* moral reason to choose greater over lesser future wellbeing in NInA cases. From a moral point of view, we should care about InA but not NInA reasons.^12^

If we hold the Great Difference view, we should choose gene editing in **Preventing a Genetic Problem** and pregnancy detection in **Preventing Birth Defects** even if these would be much less likely to prevent serious health problems than the alternative options. However, Parfit rejected this view. When he reflected on parallel InA and NInA cases, particularly those affecting large numbers of future individuals like **Preventing Birth Defects**, they seemed to him intuitively equally important. This gave rise to what he called:

**The No-Difference view:**^13^ There is equal moral reason to choose greater over lesser future wellbeing in NInA and InA cases. From a moral point of view, InA and NInA reasons should be treated symmetrically.

The No-Difference view explains why it would be wrong to deplete natural resources or choose to conceive a child who will have much lower wellbeing. It suggests that in **Preventing a Genetic Problem** or **Preventing Birth Defects**, we should choose whichever option would bring about the best outcome or be most cost-effective. But there are some challenges, too, for this view. In particular, the reasons that apply in NInA cases seem different to those that apply in InA cases. Parfit initially referred to the former reasons as “impersonal” (since there is no harm or benefit to a specific individual), and as such, they might appear mysterious. For example, Rebecca Bennett has claimed that the impersonal reasons are “abstract and difficult to comprehend.”^14^

Furthermore, we normally think that we have strong reasons to avoid harm to existing people. For example, it is usually thought to be permissible to override parents’ wishes in order to treat or prevent serious disabling health conditions in a child (for example, if parents’ refusal of treatment risks significant harm to the child).^15^ However, the ndv suggests that we have equally strong reasons to use reproductive technology to select children without those conditions. That might be thought to generate uncomfortably eugenic implications.^16^ While some philosophers have endorsed a principle of Procreative Beneficence (according to which parents have an obligation to choose to conceive children with greater predicted wellbeing), they have not generally suggested that this principle is as strong as a general Beneficence principle.^17^ A compromise view has seemed attractive to a number of philosophers.^18^ We could call this:

**The Some Difference (Two-Tier) View**:^19^ There is stronger moral reason to choose greater over lesser wellbeing in InA than in NInA cases. From a moral point of view, we should care more about InA than NInA reasons.

The Some Difference view is also sometimes labeled a Two-Tier View, since it implies that the reasons that apply to cases like these have a higher (InA) and lower (NInA) form.^20^

### 3 Problems with the Two-Tier View

The Two-Tier View is intuitively appealing. However, it gives rise to some further problems.

### 3.1 Indeterminacy

If InA reasons are more weighty than NInA reasons, it is practically important to know the magnitude of this difference. Is it small or very large? For example, if InA reasons are more than twice as weighty as NInA reasons, that would favor the first (InA) option in our imagined **Preventing a Genetic Problem** and **Preventing Birth Defects** choices. If InA reasons are less weighty than this, we should choose the second options.

However, to date, none of the philosophers who have defended a Two-Tier View have been able to clarify the magnitude of the difference between InA and NInA reasons.^21^ Jeff McMahan has observed that not only is the difference between the tiers hard to pin down; it also appears to be variable. In some cases, the difference appears intuitively to be “very slight,” while in others “there is a vast asymmetry.” He asked:

Why is the comparative strength of person-affecting considerations greater in some instances than in others? And how are the two types of consideration to be integrated into a unified account of our moral reasons? I cannot answer these questions.^22^

As McMahan reflects, it is unclear how we could arrive at the relative weight of these. This might be merely an epistemic problem. However, potentially, the problem is deeper – arguably these are different types of reasons, and there is no precise way of comparing their strength.

### 3.2 Manipulation

If reductions in wellbeing in NInA cases are less morally important than in InA cases, that may suggest some practical strategies. For example, some philosophers have argued that, depending on the approach taken, gene therapy (or mitochondrial replacement for genetic disorders with mitochondrial inheritance) may be either InA or NInA.^23^ If that is right, risky options in gene editing could be deliberately introduced in such a way that they are non-individual affecting.^24^ Or consider the following:

**Manipulating reasons**

A pharmaceutical company becomes aware that one of its best-selling medications is associated with rare teratogenic side effects. The company decides to mitigate the problem by combining the medicine with a harmless second agent that has the side effect of altering the timing of ovulation. As a consequence, any children who develop malformations related to the drug will be different from children who would have been conceived if the mothers had not taken the medicine.^25^

But it is hard to see that such manipulation would really be an improvement on the approach taken to prescribing drugs in pregnancy, artificial reproduction, or gene editing, or that it could be used as a justification for risks that would otherwise not be permitted.

In Section 3.3, I consider the suggestion that some genetic alterations might result in non-individual-affecting cases, while others would not. However, if we think that large changes in phenotype and/or changes affecting the brain will result in NInA cases, that may lead to the counterintuitive conclusion that it is more morally important to make *smaller* rather than larger genetic changes or more important to use gene therapies for conditions that do not affect the brain. The benefits would count for more.

On the other hand, if we are concerned to prevent harm (for example from off-target mutations with gene editing), it may be that we should deliberately choose to make *larger* genetic alterations or genetic changes that will affect the brain since these would be more likely to be NInA cases (and therefore any adverse effects matter less). This is hard to accept.

### 3.3 Uncertainty

Next, in at least a subset of cases affecting future individuals, it may be uncertain whether or not individuals are affected. There are several different reasons for this.

#### 3.3.1 Conception Uncertainty

Delays in the timing of conception would lead to a different combination of sperm and egg. However, with short delays, it is theoretically possible, even if unlikely, that the same sperm and egg would combine.^26^

For example, imagine that the delay in ovulation in **Manipulating reasons** is one day. That will mean that in some cases, parents do not have sexual intercourse at a time when there is an ovum in the fallopian tube. Yet in other cases, conception from the same episode of intercourse would still be possible.

For any individual case it will be impossible to determine whether it is InA or not.^27^

The effect is even more complicated for population-level cases like **Preventing Birth Defects**. While pregnancy detection might appear to be an InA case, some women (whose fetuses are affected by malformations) may choose to terminate the pregnancy, others (who are not currently pregnant) may be influenced by the pregnancy detection program to change their plans to conceive or their use of birth control. Any resulting difference in the incidence of malformations is therefore likely to include a mix of some InA and some NInA cases. Yet, the numbers of each of these will be unknown.

#### 3.3.2 Counterfactual Uncertainty

When we are considering individual cases, whether they count as InA depends on whether (if we had made a different choice) the same individual would have existed. But there may be multiple different counterfactuals, in some of which the same individual exists, and in others of which they do not.^28^

For example, in **Preventing a Genetic Problem**, the couple who are carriers of a genetic disorder have a single affected embryo. Gene editing was regarded as InA because it was implied that without gene editing, the resulting child would have been born with the genetic disease. Yet that is not the only possible alternative (or even the most likely).

For some couples, the option of gene editing will lead them to embark on ivf at a particular time and in a particular clinic. This will almost inevitably mean that the child so conceived will be different from a child that they would have conceived had they not received or responded to the offer. Next, for many couples who have carriers of a serious genetic disorder, if, following IVF and pre-implantation genetic diagnosis, they only have an affected embryo, the most common choice would be to attempt another cycle of artificial conception in the hope of having an unaffected embryo the next time.

Douglas and Devolder have argued that for these reasons in most real-world cases, gene editing will be NInA.^29^ However, whether or not these alternative possibilities change our evaluation of the nature of the case further depends on our view about counterfactuals. On one view (the Closest World view),^30^ to determine whether an individual is benefited or harmed, we should compare the actual world with the nearest possible world in which an action had not occurred. On this account, as Douglas and Devolder argue, many examples of gene editing will not lead to comparative benefit or harm. The most likely alternative (to the gene-edited child) is that the child does not exist. But there are problems with Closest World counterfactual accounts of harms,^31^ and there are alternative accounts according to which we should compare the actual world with the *salient* alternative. According to Justin Klocksiem, this alternative is “one in which the relevant agent does what would intuitively be thought of as refraining from action.”^32^ On such a view, the salient alternative to gene editing would be to not perform the gene editing. This may imply, contra Douglas and Devolder, that the majority of cases of gene editing *are* InA.^33^

My aim here is not to resolve wider debates about which is the correct account of counterfactual harm. Rather, I merely point out that uncertainty about which counterfactual is the relevant one could yield challenges for applying a Two-Tier View. Furthermore, it is worth noting, that even if we decide which account of counterfactuals is correct, there may be residual uncertainty. For example, it may not be obvious which course of action is the most likely in the absence of gene editing. If we are interested in the salient counterfactual, it may not be clear whether we should focus on the absence of a specific act (gene editing), or a cluster of acts (the offer of ivf with gene editing).

#### 3.3.3 Identity Uncertainty

Even if we decide that the correct counterfactual is that an individual exists without gene editing, that does not guarantee that this would be a NInA case. Gene therapies might result in smaller or larger changes to the genotype. If those genetic changes were at least as great as the genetic differences between siblings, on some views of identity they would result in a different individual being born (and hence a NInA case).^34^ But smaller genetic changes might also result in a different individual being born if they had a sufficient effect on the phenotype. One possibility is that genetic changes that affect the brain would result in a different individual (NInA), whereas merely somatic effects (without affecting the brain) result in the same individual being born. But genes can have widespread effects even if their main influence is on the body.

There is a tri-fold challenge. To determine whether a particular instance of gene editing would affect the identity of the resulting child, we need to know what degree of change is sufficient. Second, there is a problem of prediction of the effect of genetic change on an individual’s life. It may often be uncertain as to what degree of change would occur. Finally, since there is a spectrum of genetic and phenotypic change, it is inevitable that there will be some cases that fall into the vague boundary zone between InA and NInA cases. How should we respond to these?

### 3.4 Intransitivity

Parfit, in some of his later writing, rejected the Two-Tier View.^35^ One of his main reasons was a concern that such views generated a further problem to the ones listed above. He argued that the view generates an unacceptable intransitivity of choices. For example, consider the following.

**Embryo Choices**

A couple are choosing to implant two embryos. Genetic testing has indicated the projected lifespan of the embryos. They have the option of genetically modifying one of the embryos, though this will require selecting a third unmodified embryo.^36^

Option 1: Embryo A (70 years) and Embryo B (40 years)

Option 2: Modified Embryo B (80 years) and Embryo C (10 years)

Option 3: Modified Embryo C (50 years) and Embryo D (20 years)

An evaluation of these options might yield the following

**Table:** 

	Non-comparative (NInA) benefit (total years of life)	Comparative (InA) Harm	Comparative (InA) Benefit
Option 1	110	-40 (1 vs 2)	0
Option 2	90	-40 (2 vs 3)	+40 (2 vs 1)
Option 3	70	0	+40 (3 vs 2)

If we evaluate these in pairwise comparisons, Option 2 is better than option 1 (20 fewer total years of life, though Embryo B will live for 40 years longer), option 3 is better than option 2 (20 fewer total years of life, though Embryo C will live for 40 years longer), but on reflection, option 1 would have been better than option 3 (40 more years of life, and no one is worse off).

Note that the concern is not that intransitivity is inevitable. A simultaneous comparison of all options might generate the conclusion that a single option is superior (option 3), assuming comparative (InA) benefits are given more weight than non-comparative (NInA) benefits. However, this may only follow in the “salient counterfactual” view. It is not clear that it is possible to have two opposing counterfactuals in a Closest World counterfactual account. The point is that the evaluation of comparative benefit requires the determination of the relevant counterfactual. Which we choose will generate a different answer in terms of comparative benefits or harms. At the very least, this seems to generate a puzzle for how to apply the Two-Tier View.

## 4 The Two-Tier Deontic View

The potentially paradoxical evaluation in **Embryo Choices** arises because of the importance of counterfactuals in identifying whether a particular choice is individual affecting or non-individual affecting. One key factor is whether we are determining the goodness of outcomes or the rightness of acts. Parfit argued that it is extremely difficult to accept that the absolute *value of the outcome* in cases like this could depend on which comparison you are making. For this reason he rejected what we could call:

***Two-Tier Telic views*:** This is a view according to which the outcome of InA choices is of greater value than the outcome of NInA choices with equivalent effects on wellbeing.^37^

However, although the value of outcomes cannot change, it is plausible to think that the reasons that we have to act, and therefore the *rightness of acts*, does depend on the options that are available to us. Consider the following example:

**Death at sixty**

Geneticists have recently identified a rare genetic disorder that is asymptomatic until it causes sudden death at age 60. There is no cure for the gene, but with costly medical treatment, it is possible to postpone the effect for a short period.

Jack has just turned 60 and has been identified with the gene. I am Jack’s doctor. With treatment, I could enable him to live to age 61. However, there is limited funding available for treatment of this disorder. For the same cost, population screening could identify a couple who are carriers for this gene, and enable them to undertake ivf and embryo selection. If this is chosen, FutureJane (who will live for 80 years) rather than FutureSally (who would live for 60 years) will come into existence.^38^

To many people, I suspect, my moral reason in **Death at Sixty**, to prolong Jack’s life for 1 year is considerably stronger than my reason to cause one of two possible future people to exist.

If we think in this way, our beliefs are about what it is right to do, not about what would be best overall. Indeed, we might acknowledge that saving Jack is right even though it would result in the worse overall outcome. Parfit summarized this: “We might believe that … we ought to give some benefits to actual people rather than giving some greater benefits to people who are now merely possible and who, if we did not give them these benefits, would never exist.”^39^

In **Death at Sixty**, it appears that the reason to make an InA choice is twenty times stronger than the reason to make a NInA choice. If this applied broadly, it would have profound implications for application of the Two-Tier View. However, the moral reason to make an InA choice might vary with the circumstances. Here is a variation on the case that might make this clear.

**Future Death at Sixty.**

Geneticists have recently identified a rare genetic disorder that causes sudden death at age 60. There is no current cure or treatment for the disease, but it is anticipated that with costly and time-consuming research, a medical treatment will be developed to postpone the effect for a short period.

There are limited resources available for this disorder. If those resources are spent on medical treatment, a (not yet conceived) FutureJack will be able to be treated and live to age 61 rather than dying at age 60.

For the same cost, population screening could identify a couple who are carriers for this gene, and enable them to undertake ivf and embryo selection. If this is chosen, FutureJane (who will live for 80 years) rather than Future Sally (who would live for 60 years) will come into existence.

Intuitively, our response to **Future Death at Sixty** may be quite different to our response to **Death at Sixty**. If we are making decisions in relation to individuals who will all exist in the future, it is much less clear that we have a strong reason to favor Jack.^40^ The reason to benefit FutureJack does *not* seem stronger than our reason to make the corresponding NInA choice (or at least not to the same extent). How can our moral reasons vary in this way?

As already indicated, this is not a function of the outcome; the outcome in the two variants are the same. Nor can we explain this in terms of the individuals who might be counterfactually benefited or harmed. Both Jack and Future Jack could complain that they have been harmed if they were not treated.

Rather, if there is a greater moral reason to treat Jack in **Death at Sixty**, it potentially arises from the special obligations that agents have to some individuals. Those obligations might arise from professional roles (for example if one were Jack’s doctor), or from relationships. Those roles or relationships generate InA reasons that in some cases justify agents making choices that benefit (or avoid harming) an individual even at the cost of a worse outcome overall. But these relationships would not usually apply to individuals who do not yet exist. Where we do not have special obligations or relationships, we have symmetrical moral reason to make InA and non-InA choices. This suggests the following:

**Two-Tier Deontic View** (TTDV): A view according to which, the moral reason to make an InA choice is stronger than the reason to make equivalent NInA choices, if and to the extent that agents have special obligations to benefit or prevent harm occurring to existing or future individuals

It may help to point out that the upper tier on this view is a subset of all individual-affecting cases. It applies to individuals who will be counterfactually benefited or harmed, but not to all such individuals. It is therefore closer to the No-Difference view than other Two-Tier views. However, it could identify a large difference between InA and NInA choices in some cases.

In the next section, I will look at the practical implications of the ttdv. But before that, I will make some general observations. First, the deontic version of the Two-Tier View has several advantages. It aligns with the nature of the reasons that apply in individual-affecting compared with non-individual-affecting cases. Where the wellbeing of the future individuals who exist is the same, the difference (if there is a difference) cannot be a function of the outcome. Arguably, it arises from our moral obligations and reasons to cause benefit or prevent harm to particular individuals with whom we have special relationships.

Second, understanding the nature of the reasons may help to explain different intuitive responses to cases such as those described at the start of this paper or to **Death at Sixty**. Those who are drawn to consequentialism (particularly act consequentialism) are likely to see no difference between InA and NInA cases because the wellbeing level is the same. But those inclined to non-consequentialist theories, will likely want to give extra weight to obligations to specific individuals.

Third, we could understand the different reasons that apply to choices that affect people who will or may exist in the future as moral vectors that sometimes converge, and sometimes pull apart.^41^ Problems of indeterminacy would remain challenging, but they are familiar problems in pluralistic value systems. Intransitivity would be less of a problem. Our moral obligations and duties *are* affected by the options that are available to us. It is not necessarily paradoxical or even unusual to find that a particular choice is permissible where an alternative does not exist, but impermissible where an alternative is available to us.

Fourth, as the versions of **Death at Sixty** illustrate, our duties may be contextual and change depending on the nature of the recipient and our relationship to them. This explains how, as McMahan observed, the relative strength of the moral reasons to choose in InA cases could change depending on the case. It also means that some of the concerns relating to manipulation and uncertainty are less problematic. I will explore that further in the next section.

Finally, I have described this view as a variant of a Two-Tier View (one in which the higher tier applies to a subset of cases in which special relations give rise to deontic reasons). But we could describe the view in a different way as a variant of the No-Difference view in which the general wellbeing related reasons that apply in InA and NInA cases are of the same magnitude, but where the reasons that arise from special relations are stronger (or apply only) in InA cases.^42^

## 5 Decision Making and Practical Implications

### 5.1 Public Health Policy and Preventing Birth Defects

**Preventing Birth Defects** is an example of a choice between InA and NInA actions occurring at a population level. How should policymakers decide about such cases?

It seems relatively clear that the primary ethical duty of policymakers would be to promote the health and wellbeing of current and future individuals in the community, and to avoid the negative consequences of medical treatments.^43^ They should consider carefully the overall effect of different policies. However, policymakers do not have individual-affecting duties that generate partiality or obligations to specific future individuals.^44^ On the contrary, we might think that their role requires impartiality. On the Two-Tier View that I have defended, they should choose largely or entirely based on which option (or combination) would avoid most future birth defects or be less costly. For example, in **Preventing Birth Defects**, if, as suggested, it would prevent twice as many birth defects, decision makers should prefer pregnancy prevention. Indeed, they should do so, even if the difference in effectiveness were much smaller. A separate reason for treating policy-level decisions in this way is the suspicion that any such large-scale choices will tend to have a wide ripple effect on people’s lives and as a consequence change the timing of conception (i.e., be NInA). However, on the ttdv, we do not need to depend on this to justify choosing the most effective option.

This points to several advantages of the ttdv for decision making. When making policy-level decisions that will affect future (not yet existing) individuals, we can avoid some of the challenges identified in Section 3. For such cases, we do not need to work out how to weigh InA against NInA effects, since they can be treated similarly. The different forms of uncertainty (conception uncertainty, counterfactual uncertainty, identity uncertainty) are not a problem.^45^ Manipulation of harm by making cases NInA (Section 3.2) would also be of no benefit.

### 5.2 Fertility Treatment and Gene Editing

Those who are considering policy relating to fertility treatment or gene editing have similar ethical responsibilities to those considering public health policy. They have obligations to conceiving couples and to wider society to avoid health problems in the resulting children. For example, Ben Saunders refers to such other-regarding reasons as providing a duty of generalized procreative non-maleficence.^46^ On such a basis, policymakers have individual affecting duties to society and to the parents of the children conceived as a result of such treatment (whether or not the children are counterfactually benefited or harmed). If considering whether to permit or prohibit gene editing, they should (on the view that I am defending), treat it similarly regardless of whether it is believed to be individual affecting.

Health professionals or scientists engaged in reproductive healthcare or science have similar ethical responsibilities to policymakers. That would again suggest a parallel approach to InA and NInA actions (and diminish some of the epistemic challenges raised in Section 3). This would undermine the “Benefit Argument” in favor of gene editing rather than selection.^47^ Some have claimed that on a Two-Tier View, we have stronger moral reason to edit out disease than to perform genetic selection. However, on the ttdv that I have described, this moral preference would usually not apply.

But perhaps such health professionals have an *additional* duty to children who are harmed by treatment that they have provided, on the basis of an ethical principle of non-maleficence. That might give the professionals concerned more reason to avoid the risk of harms in InA forms of gene editing. Some forms of legal liability might also track individual-affecting reasons. So, health professionals may be liable for negligence to the children conceived for (counterfactual) harms in InA cases. Yet, that may not necessarily generate a strong asymmetry. In non-individual-affecting cases they will also be liable to the parents and carers of children born with problems attributed to ggt. Furthermore, it is not clear from an ethical perspective that if a health professional or researcher were to negligently cause off-target mutations in a NInA case, that they should be any less blameworthy. In a highly relevant recent UK court case, a doctor was found to be negligent in relation to failure to advise pre-conception folate for a child subsequently born with spina bifida, even though it was acknowledged that, had appropriate advice been given, the plaintiff would not have been born (a delay in conception to increase folate intake would have led to the parents conceiving a different child).^48^

Parents, however, potentially have a different perspective. That is because they have ethical obligations to their existing children arising from their relationship to them. They may also anticipate the special relations that they will have with future-existing children. Parents may have more moral reason to choose an InA form of gene therapy than embryo selection because they have special reasons of partiality to their children and because this would benefit a specific future child.^49^ On the other hand, parents also have to weigh more heavily the possibility that complications of the gene editing would harm their child. That may give them greater reason to choose embryo selection, particularly where the risks of gene editing are uncertain or unknown.

Although it does not resolve all of the uncertainty problems identified above, on the Two-Tier Deontic View, parents would have a stronger reason to make an InA choice in cases where there is an existing individual who would be counterfactually benefited or harmed.

One complication is that these individual-affecting reasons may be partly a function of moral status and parents’ evolving relationship with their offspring.^50^ That would imply, for example, that parents have stronger reason to pursue gene therapy for an older child than for a newborn, or a fetus, or a conceptus. If the embryo has no moral status, then parents may not have a special duty to the embryo that is different from their duty to other possible children that they could conceive. While I am sympathetic to that view, I do not propose to resolve here the nature of the moral status of the embryo or fetus, but rather to point out that questions of InA duties may supervene on these separate questions.

A second complication, particularly relevant to gene editing, is that (as noted in Section 3.3) interventions in relation to the early embryo might alter the identity of the subsequently existing child. Where such an effect is predicted, that would diminish the deontic reason to gene edit. (Where it is uncertain, the problem of ‘identity uncertainty’ may make it difficult to know what duties parents have to gene edit.)

## 6 Objections

Although I have suggested that the ttdv provides a plausible and intuitive basis for our ethical obligations, and points to some practical advantages, there are some predictable objections.

### 6.1 Negative Consequences

One objection will be that when our obligations to specific individuals lead to priority to InA choices, that will lead to much worse overall consequences.

It is inescapable that adopting a Two-Tier View will lead to worse consequences. How great a concern this is will depend on how strong we take our InA duties to be and how widely affecting are our choices. In Section 5, I suggested that decisions relating to future individuals taken at a policy level should usually treat InA and NInA choices symmetrically. This will potentially diminish any negative effects and mean, for example, that policymakers should regulate fertility treatment or gene editing in a way that will lead to the greatest wellbeing of the resulting future children. However, some choices may involve trade-offs between current and future individuals. For example, imagine that in **Preventing a Genetic Problem**, policymakers were considering a third option:

**Preventing a Genetic Problem – Postnatal prevention^51^**

Option 3: In the third option, children who are affected by a genetic disorder are identified by newborn screening and commenced on a highly expensive form of gene therapy. This therapy diminishes (though does not eliminate) the effect of the genetic disorder.

For policymakers, but perhaps even more so for doctors and for parents, it is plausible to think that they *do* have individual affecting obligations to existing newborn infants.^52^ That may imply that if forced to choose, there is stronger moral reason to choose postnatal prevention over embryo selection or gene editing, even if the latter two options would prevent many more cases or were much less costly.

This appears to be an inevitable consequence of the ttdv, and those who defend it need to bite this bullet. I do not have space here to explore the wider implications, except to note that it will be important to establish the limits of justified partiality towards current individuals. In choices involving trade-offs between current individuals (to whom policy makers owe specific duties) and future individuals (e.g., in relation to the environment or climate), it may be important to have mechanisms to ensure that NInA reasons are given sufficient moral weight.

### 6.2 Fixed Duty to Future Individuals

Some may respond to the above arguments by claiming that policymakers in **Preventing Birth Defects** *do* have obligations to benefit or prevent harm to specific future individuals who will be affected by their decisions. The suggestion would be that such an obligation arises inherently from the nature of counterfactual harms and benefits. It does not arise in NInA cases because of non-identity. For example, Hope and McMillan argue, in a case that parallels **Preventing Birth Defects**, that there is stronger reason to avoid 100 InA cases of future deafness than 100 NInA cases.^53^ Alternatively, the claim might be that even if policy makers do not now have duties to future individuals, they *will have* such duties once those children are born (and have been harmed or benefited).

It is difficult to either refute or defend such a claim without begging the question. It is clear that the difference between InA and NInA cases is the presence or absence of an alternative in which individuals exist. The question is whether that mere fact generates a corresponding moral reason or obligation. In part that will depend upon our theory of moral reasons. It is relatively familiar that some reasons arise from relationships and roles. They may, therefore, also vary in strength.

If we think that there is a difference between **Death at Sixty** and **Future Death at Sixty**, this implies that our moral reasons are not fixed by the nature of counterfactual harms. Likewise, if there is a stronger moral reason to perform postnatal prevention rather than gene editing in **Preventing a Genetic Problem**, that again suggests that the strength of our moral reason is not (simply) a function of counterfactual harm. However, once more, intuitive responses to these cases may differ.

I have suggested that the ttdv provides a more plausible response to cases like **Death at Sixty** and avoids some of the challenges of the Two-Tier Problem. However, if we reject the ttdv but wish to retain a Two-Tier View, the problems identified in Section 3 will remain.

### 6.3 Presentism

Lastly, one obvious difference between **Death at Sixty** and **Future Death at Sixty** is that Jack is a currently existing individual, whereas FutureJack is a future person. One question might be whether the variable strength of moral reasons in the ttdv is simply a function of co-existence in time. Parfit’s description of Two-Tier views, in his final published paper, included the speculation that perhaps “we ought to give some benefits to presently existing people rather than giving some greater benefits to future people.”^54^ Two-Tier views that incorporated this principle might be accused of bias against future generations.

The ttdv as I have described it will often have presently existing individuals in the higher tier and future individuals in the lower tier. That is because the roles that I have suggested would give rise to stronger moral reasons to benefit or avoid harm (e.g., being a parent or a doctor) will usually apply to existing individuals and not to future ones. However, the ttdv will not necessarily give preference to existing individuals. For example, we could imagine that in **Death at Sixty**, the agent were not Jack’s doctor, but an anonymous philanthropist who is deciding whether to fund the life-prolonging treatment or genetic selection. If the philanthropist has no relationship to Jack, it is plausible to claim that she has equal moral reason to fund InA or NInA choices, and in the case at hand, has much more reason to fund genetic selection (since that will lead to 20 years longer life). The ttdv may also (in some circumstances) identify a stronger moral reason to benefit a future not-yet-conceived individual than confer a NInA benefit. For example, as noted in Section 5.2, parents may have special moral obligations to their not-yet-conceived children.^55^

## 7 Conclusions

In this paper, I have outlined some of the challenges in applying a Two-Tier View as well as a potential way of responding to these. I have not sought, directly, to defend such a view. It is clear that the problems of indeterminacy, manipulation, uncertainty, and intransitivity could be avoided by adopting either the Great Difference or No-Difference View. However, if we are drawn to the compromise view, there is a need to look for ways to address the Two-Tier Problem. If we can find such solutions, that would make the compromise more attractive.

I have developed and defended a view that captures the nature of the different moral reasons in individual-affecting and non-individual-affecting cases. If there is a difference between these types of case, it is most plausible that this arises from our obligations as agents rather than from the value of outcomes. The Two-Tier Deontic View has the advantage of explaining how the difference between InA and NInA choices can be considerable in some situations, but negligible or absent in others. It also avoids (to at least a degree), some of the problems of a fixed or telic Two-Tier View. Cases in which it is unclear whether or not individuals will be affected by our decisions or where large numbers of individuals will be affected are typically those in which there are not strong obligations to the individuals who will exist. At the other extreme, where there *is* an identifiable individual who will be affected, decision makers (particularly parents or health professionals) may indeed have duties to the child or future child that would justify them making a choice that will lead to lower wellbeing overall.

I started this paper with a description of potential decisions needing to be made between InA and non-InA options in two different situations. Philosophical discussion of the non-identity problem can seem abstract and divorced from real-life choices. Yet, both of these examples were inspired directly by situations that I encountered as a health professional. On my account, the ttdv could help with practical decision making. I do not claim that this view solves all the problems of decision making about future individuals. Duties to future individuals potentially vary between decision makers, and with moral status. We will have to decide how to weigh deontological against consequentialist considerations and establish the limits of moral partiality towards individuals with whom we have special relationships.

Yet, as outlined, some cases that involve choices between future individuals who will or may exist seem more tractable. By distinguishing these, we may at least make some progress.

## Footnotes

1 The widely used anti-epileptic sodium valproate causes birth defects in approximately 1 in 10 fetuses whose mothers take it during pregnancy, while approximately 4 in 10 have learning or developmental problems, https://epilepsysociety.org.uk/about-epilepsy/sodium-valproate/concerns-around-sodium-valproate.

2 The use of highly effective contraceptives may reduce the total number of children born to the women taking the medicine, or it may simply alter the timing of conception (i.e., delaying conception until they are taking a different medicine). For the sake of this paper, I will assume the latter effect, in order to set aside effects on the numbers of individuals conceived.

3 D Parfit, *Reasons and persons* (Oxford: Oxford University Press, 1984): 351–80.

4 Thomas Douglas and Katrien Devolder, “Gene Editing, Identity and Benefit,” *The Philosophical Quarterly* 72, no. 2 (2021): 305–25, https://doi.org/10.1093/pq/pqab029. A similar argument has been made in favor of certain forms of mitochondrial replacement. Wrigley and colleagues claim that there is a stronger obligation to use techniques that are individual affecting (compared to ones that are non-individual affecting). A. Wrigley, S. Wilkinson, and J. B. Appleby, “Mitochondrial Replacement: Ethics and Identity,” *Bioethics* 29, no. 9 (Nov 2015): 631–38, https://doi.org/10.1111/bioe.12187.

5 Another practical example where this problem might arise is in relation to interventions for severe congenital infections. See K. Doolabh et al., “Zika, contraception and the non-identity problem,” *Dev World Bioeth* 17, no. 3 (Dec 2017): 173–204, https://doi.org/10.1111/dewb.12176.

6 Jeff McMahan, “Causing People to Exist and Saving People’s Lives,” *The Journal of Ethics* 17, no. 1–2 (2013): 5–35, https://doi.org/10.1007/s10892-012-9139-1.

7 Jeff McMahan, “Asymmetries in the morality of causing people to exist,” in *Harming future persons: ethics, genetics and the nonidentity problem*, ed. Melinda Roberts and David Wasserman (New York: Springer, 2009), footnote 2.

8 See, e.g., Matthew Hanser, “The metaphysics of harm,” *Philosophy and Phenomenological Research* 77, no. 2 (2008): 421–50. Seana Valentine Shiffrin, “Wrongful life, procreative responsibility, and the significance of harm,” *Legal Theory* 5, no. 2 (1999): 117–48. Molly Gardner, “A harm based solution to the non-identity problem,” *Ergo: An Open Access Journal of Philosophy* 2 (2015): 427–44.

9 Parfit (1984), *op. cit*., note 2, 356.

10 This view is sometimes referred to as the person-affecting restriction (Parfit 1984), op. cit., note 2, 394) or the strong narrow Person affecting principle, Derek Parfit, “Future People, the Non-Identity Problem, and Person-Affecting Principles,” *Philosophy & Public Affairs* 45, no. 2 (2017): 118–57, 118, https://doi.org/10.1111/papa.12088.

11 Tony Hope and John McMillan label this the “Great Difference View.” Tony Hope and John McMillan, “Physicians’ Duties and the Non-Identity Problem,” *American Journal of Bioethics* 12, no. 8 (2012): 21–29, https://doi.org/10.1080/15265161.2012.692432.

12 For advocates of this view, see Jan Narveson, “Moral problems of population,” in *Ethics and population*, ed. Michael D. Bayles (Cambridge, Mass: Schenkman Pub. Co, 1976). R. Bennett, “The fallacy of the principle of procreative beneficence,” *Bioethics* 23, no. 5 (May 9 2008): 265–73, https://doi.org/10.1111/j.1467-8519.2008.00655.x. David Heyd, “Parfit on the Non-identity Problem, Again,” *Law and Ethics of Human Rights* 8, no. 1 (2014): 1–20.

13 Parfit (1984), *op. cit*., note 2, 367.

14 Bennett, *op. cit*., note 12, 5. In later work, Parfit used instead a “wide person affecting principle” to ground the No-Difference view (Parfit 2017, *op. cit*., note 10, 123–24). According to this principle, it is better to benefit people more by bringing them into existence with greater predicted wellbeing.

15 D. S. Diekema, “Parental refusals of medical treatment: the harm principle as threshold for state intervention,” *Theoretical Medicine and Bioethics* 25, no. 4 (Jan 1 2004): 243–64; Lynn Gillam, “The zone of parental discretion: An ethical tool for dealing with disagreement between parents and doctors about medical treatment for a child,” *Clinical Ethics* 11, no. 1 (2015): 1–8, https://doi.org/10.1177/1477750915622033sx.

16 Bennett, *op. cit*., note 12.

17 Julian Savulescu and Guy Kahane, “The moral obligation to create children with the best chance of the best life,” *Bioethics* 23 (2009): 274–90, 277, https://doi.org/10.1111/j.1467-8519.2008.00687.x.

18 For example, Hope and McMillan, *op. cit*., note 11, Julian Savulescu, Melanie Hemsley, Ainsley Newson and Bennett Foddy, “Behavioural genetics: Why eugenic selection is preferable to enhancement,” *Journal of Applied Philosophy* 23, no. 2 (2006): 157–71. Jeff McMahan, “Wrongful life: paradoxes in the morality of causing people to exist,” in *Bioethics*, ed. J. Harris (Oxford: Oxford University Press, 2001). Noam J. Zohar, “Prospects for “genetic therapy” – can a person benefit from being altered?,” *Bioethics* 5, no. 4 (1991/10// 1991): 275–88, https://doi.org/10.1111/j.1467-8519.1991.tb00168.x. G. Arrhenius, “The person-affecting restriction, comparativism, and the moral status of potential people,” *Ethical Perspect* 10, no. 3–4 (2003): 185–95, https://doi.org/10.2143/ep.10.3.503884.

19 When he first described the non-identity problem, Parfit noted that there were three possible views: “It might make all the difference, or some difference, or no difference” (Parfit, *op. cit*., note 2, 363). Hope and McMillan call this a “midway view,” *op. cit*., note 11

20 Derek Parfit, On what matters, 2 vols. (Oxford: Oxford University Press, 2011): 219.

21 Parfit, in On What Matters, considered, for the sake of argument that InA might be twice as weighty as NInA reasons (p. 221). However, he did not provide any rationale for this, and later rejected the two-tier view. *Parfit, On what matters, 231*.

22 McMahan, *op. cit*., note 18, 475. The argument developed in this paper offers a response to McMahan’s question.

23 Alonso and Savulescu have argued that different forms of germline gene editing may be either InA or NInA and this may be relevant to the evaluation of their permissibility. Marcos Alonso and Julian Savulescu, “He Jiankui’s gene-editing experiment and the non-identity problem,” *Bioethics* 35, no. 6 (2021): 563–73, https://doi.org/10.1111/bioe.12878.

24 The same could apply to fertility treatment. E.g., see D. Wilkinson et al., “Double trouble: should double embryo transfer be banned?,” *Theor Med Bioeth* 36, no. 2 (Apr 2015): 121–39, https://doi.org/10.1007/s11017-015-9324-x, http://www.ncbi.nlm.nih.gov/pubmed/25813034.

25 Even small delays in the timing of conception would potentially mean that a different spermatozoan would combine with the ovum, and hence lead to a genetically different individual being conceived. Parfit, *op. cit*., note 2, 352.

26 Parfit, *op. cit*., note 2, 352.

27 Sperm from an ejaculate can remain viable in the body for up to 5 days following intercourse. Ova are usually only viable for 12–24 hours after ovulation. C. R. Weinberg et al., “The Probability of Conception as Related to the Timing of Intercourse Around Ovulation,” *Genus* 54, no. 3/4 (1998): 129–42, http://www.jstor.org/stable/29788567.

28 Pitcovski and Peet have recently discussed wider indeterminacy problems with counterfactual accounts of harm. Eli Pitcovski and Andrew Peet, “Counterfactuals, indeterminacy, and value: a puzzle,” *Synthese* 200, no. 1 (2022/02/28 2022): 51, https://doi.org/10.1007/s11229-022-03464-w. Here, I focus only on the question of whether or not a particular action is InA or NInA. As another example of uncertainty, Patrick Tomlin has argued that some cases involving multiple actions are “impure non-identity cases.” Patrick Tomlin, “The Impure Non-Identity Problem,” in *Ethics and Existence: The Legacy of Derek Parfit*, ed. Jeff McMahan et al. (Oxford University Press, 2022). They comprise some actions that are InA, though the collection of actions are NInA. He argues that such cases provide support for the No Difference view, though the view that I develop below would also be able to deal with such cases.

29 Douglas and Devolder, (2021) *op. cit*., note 4. They concede that cases like **Preventing Genetic Problems** could be InA in situations where the couple are committed to implanting an affected embryo in the absence of gene editing. Alonso and Savulescu raise similar concerns about the difficulty of identifying the relevant counterfactual, *op. cit*., note 23.

30 Nathan Hanna, “Harm, Omission, Preemption, Freedom,” *Philosophy and Phenomenological Research* 93, no. 2 (2016): 251–73, https://www.jstor.org/stable/48578730.

31 Duncan Purves, “Harming as making worse off,” *Philosophical Studies* 176, no. 10 (2019/10/01 2019): 2629–56, https://doi.org/10.1007/s11098-018-1144-1.

32 Justin Klocksiem, “A Defense of the Counterfactual Comparative Account of Harm,” *American Philosophical Quarterly* 49, no. 4 (2012): 285–300, 294.

33 Jeff McMahan and Julian Savulescu have recently made this argument. Jeff McMahan and Julian Savulescu, “Reasons and Reproduction: Gene Editing and Genetic Selection,” *The American Journal of Bioethics*: 1–11, https://doi.org/10.1080/15265161.2023.2250288.

34 Zohar has argued that germline gene editing would in almost all cases affect identity and hence be NInA, *op. cit*., note 18.

35 Parfit, *On what matters*, vol 2 219–31. Parfit (2017), *op. cit*., note 10, 231.

36 This is adapted from Parfit’s Case 6, 2017, ibid., 228.

37 Parfit, in his writing, referred to telic and deontic versions of person-affecting (InA) principles. However, to my knowledge, he did not distinguish between different possible two-tier views.

38 This is a version of Parfit’s “Case 5,” Parfit (2017), *op. cit*., note 10, 147. McMahan discusses a series of variations of similar cases in his paper (McMahan 2012), *op. cit*., note 5, and defends the common intuition.

39 Parfit (2017, 148).

40 Ibid., 147

41 J Savulescu and E Protopapadakis, “‘Ethical Minefields’ and the Voice of Common Sense: A Discussion with Julian Savulescu,” *Conatus Journal of Philosophy* 4, no. 1 (2019), https://doi.org/10.12681/cjp.19712.

42 I am very grateful to Jeff McMahan for this suggestion. The practical implications of the alternative framing of the view would be potentially the same. One potential advantage would be that the No-Difference view (as modified) would deviate less from the two-tier view than has sometimes been thought.

43 For the purposes of the paper, I will set aside the nature or strength of this duty, since the point here is that these reasons apply symmetrically to InA and NInA cases.

44 I consider a response to this claim in the next section.

45 Cf. Tomlin *op. cit*., note 28.

46 B. Saunders, “First, do no harm: Generalized procreative non-maleficence,” *Bioethics* 31, no. 7 (Sep 2017): 552–58, https://doi.org/10.1111/bioe.12366.

47 Douglas, *op. cit*., note 3.

48 Toombes v Mitchell, [2021] ewhc 3234 (qb).

49 Others have argued that deontic reasons apply in InA but not in NInA cases. Abelard Podgorski has argued that parents do not have partial reasons (of special concern) that apply in procreative decisions. Abelard Podgorski, “Partiality, Identity, and Procreation,” *Philosophy and Public Affairs* 49, no. 1 (2021): 51–77. He rejects arguments (e.g., based on *de dicto* betterness) that parents have a special reason qua parent to bring into existence children with higher wellbeing. This supports the claim that in some cases (where there are agent-relative obligations that apply), there are stronger reasons to make an InA than a corresponding NInA choice. Tom Douglas has argued that the standard justifications for parental partiality are weaker in relation to future not-yet-conceived children (even in InA cases) than they are for existing children. Thomas Douglas, “Parental Partiality and Future Children,” *Journal of Ethics and Social Philosophy* 15, no. 1 (2019), https://doi.org/10.26556/jesp.v15i1.351.

50 Frances Kamm has made a similar argument that in InA cases, the permissibility of choices may depend on moral status and that choices affecting embryos (because they lack a certain kind of moral status) may be treated similarly to NInA choices. F.M. Kamm and F.M. Kamm, “Moral Status, Rights, and Parfit’s No-Difference View,” in *Rights and Their Limits: In Theory, Cases, and Pandemics* (Oxford University Press, 2022).

51 One real example of a genetic disorder for which this applies is Spinal Muscular Atrophy. The postnatal gene therapy, Zolgensma, is often described as the most expensive drug in the world. Angus Livingstone, Laurent Servais, and Dominic J. C. Wilkinson, “Crowdfunding for neuromuscular disease treatment: the ethical implications,” *The Lancet Neurology* 20, no. 10 (2021): 788–89, https://doi.org/10.1016/S1474-4422(21)00266-0.

52 The justification for a special obligation on the part of policymakers to *currently* existing newborn infants might be political more than ethical. (For example, it might correspond to their politically derived obligations to be (at least somewhat) partial to current citizens of a country.) If there is such an obligation, the strength of the deontic reason is likely to depend on the moral significance of the special relation, and correspondingly to be stronger for parents (based on parental relations), than physicians (based on professional duties), or policy makers. I am grateful to Jeff McMahan for this suggestion.

53 *Op. cit*., note 11, 45–46. Although they endorse the some-difference view, they appear to see the reason or duty that arises as being fixed.

54 Parfit (2017), *op. cit*., note 10, 148.

55 Tom Douglas argues that such pre-parental partial reasons are much weaker than their partial reasons towards currently existing children. *Op. cit*. note 49.
